# Focusing on the amount of immediate changes in spinopelvic radiographic parameters to predict the amount of mid-term improvement of quality of life in adult degenerative scoliosis patients with surgery

**DOI:** 10.1007/s00402-022-04667-z

**Published:** 2022-11-08

**Authors:** Yanbin Liu, Jinlong Liu, Dawei Luo, Jianmin Sun, Feng Lv, Bin Sheng

**Affiliations:** 1 Department of Orthopedic Surgery, Liaocheng People’s Hospital, Liaocheng, Shandong People’s Republic of China; 2 Department of Spine Surgery, Shandong Provincial Hospital Affiliated to Shandong University, Jinan, Shandong People’s Republic of China; 3 Department of Pain, Zibo Central Hospital, Zibo, Shandong People’s Republic of China

**Keywords:** Adult degenerative scoliosis, Spinopelvic radiographic parameters, Immediate changes, Quality of life, Mid-term improvement

## Abstract

**Introduction:**

Surgery is still an effective treatment option for adult degenerative scoliosis (ADS), but how to predict patients’ significant amount of the improvement in quality of life remains unclear. The previous studies included an inhomogeneous population. This study aimed to report the results about concentrating on the amount of immediate changes in spinopelvic radiographic parameters to predict the amount of mid-term improvement in quality of life in ADS patients.

**Materials and methods:**

Pre-operative and immediately post-operative radiographic parameters included Cobb angle, coronal vertical axis (CVA), sagittal vertical axis (SVA), lumbar lordosis (LL), thoracic kyphosis (TK), pelvic tilt (PT), sacral slope (SS), pelvic incidence (PI) and LL/PI matching (PI–LL). Quality of life scores were evaluated pre-operatively and at the final follow-up using Oswestry Disability Index (ODI) and visual analogue scale (VAS). The amount of immediate changes in spinopelvic radiographic parameters (Δ) and the amount of mid-term improvement in quality of life (Δ) were defined, respectively.

**Results:**

Patients showed significant change in radiographic parameters, ODI and VAS pre- and post-surgery, except CVA and PI. Univariate analysis showed a significant correlation between ΔTK, ΔLL, ΔCVA and the amount of mid-term improvement in quality of life, but multivariate analysis did not get a significant result. Univariate and multivariate analyses showed that ΔSVA was still a significant predictor of ΔVAS and ΔODI. The changes in the other radiographic parameters were not significant. The equations were developed by linear regression: ΔODI = 0.162 × ΔSVA − 21.592, ΔVAS = 0.034 × ΔSVA − 2.828. In the ROC curve for ΔSVA in the detection of a strong ΔODI or ΔVAS, the cut-off value of ΔSVA was − 19.855 mm and − 15.405 mm, respectively.

**Conclusions:**

This study shows that ΔSVA can predict the amount of mid-term improvement in quality of life in ADS patients. The changes in the other radiographic parameters were not significant. Two equations were yielded to estimate ΔODI and ΔVAS. ΔSVA has respective cut-off value to predict ΔODI and ΔVAS.

## Introduction

With the ageing of the population and the increase in people's demand for quality of life, ADS is gaining more and more attention [1, 2]. ADS as a special type of adult scoliosis is one of the most challenging spinal deformity, and includes a complex part of spinal diseases [3]. Patients with ADS failing to conservative treatments may choose surgery [4]. Surgery is technically challenging, such as progressive junctional kyphosis [5, 6], but operative intervention is still an effective and reasonable treatment option for ADS with some satisfactory clinical outcomes, such as relieving pain and increasing capacity for activity [4–7]. Improving patients' quality of life are the main problem that spinal surgeons have to solve. Spinal surgeons often want to know the changes in which parameters can lead to definite improvements in quality of life and especially want to know the significant amount of the improvement in quality of life.

To spinal surgeons, the easiest available parameters are radiographic parameters before and after surgery. Radiographic parameters are very important to the surgical outcomes of adult deformities [8–12]. For example, post-operative quality of life was significantly associated with some pre-operative sagittal parameters (SVA, T1 sagittal tilt), but the impact of some pre-operative radiographic parameters (SVA, T1 sagittal tilt, PT, PI–LL) on pre-operative quality of life was not strong [10]. These studies have only showed the relationship between some spinopelvic radiographic parameters and quality of life scores, but they have never quantified the changes in spinopelvic parameters and the changes in quality of life. These studies included an inhomogeneous population including with ADS and adult idiopathic scoliosis and/or other types. ADS has different pathogenesis, clinical manifestation and treatment methods compared with other types, so ADS should be studied separately.

To the best of our knowledge, there is still a paucity of study in the literature about the relation between the amount of improvement in quality of life and the amount of immediate changes in spinopelvic radiographic parameters of ADS alone. Therefore, this study aimed to get the results of this relation regarding to use the amount of immediate changes in spinopelvic radiographic parameters to predict the amount of mid-term improvement in quality of life of ADS. It will be helpful to know the immediate changes in which radiographic parameters are the most important and the more likely to lead to the significant amount of mid-term improvement in quality of life of ADS.

## Materials and methods

### Patients

We conducted a retrospective review of a single-centre database of prospectively collected patients with adult degenerative lumbar or thoracolumbar scoliosis undergoing surgery between March, 2014 and January, 2019 at the Shandong Provincial Hospital Affiliated to Shandong University. Ethical approval was obtained by the Ethics Committee of Shandong Provincial Hospital Affiliated to Shandong University. Informed consent was obtained for experimentation with patients.

Inclusion criteria included: (1) diagnosis of ADS according to the Aebi classification system of adult scoliosis (Aebi type I); (2) aged ≥ 50 years at surgery; (3) a coronal Cobb angle of major curve ≥ 30° [13]; (4) complete radiographs and questionnaires available; and (5) at least 2 years of follow-up after surgery.

Exclusion criteria included: (1) other types of adult spinal scoliosis (idiopathic scoliosis, neuromuscular scoliosis, etc.); (2) complicating with other pathology (tumour, fracture, etc.); or (3) patients with previous spinal surgery; (4) patients with reoperation, or (5) severe surgical complications, such as nerve root damage.

Medical records, radiological images and self-reported questionnaires were used to collect the data. The surgical techniques hereby have been described previously [12, 14, 15].

### Measurement of radiographic parameters

The immediately post-operative (no more than 7 days after surgery) and pre-operative anteroposterior and lateral standing radiographs with entire spine and bilateral femoral heads were analysed according to the previous studies [16, 17]. Radiographic parameters included coronal radiographic parameters (Cobb angle of major curve, CVA), sagittal radiographic parameters (SVA, TK, LL), pelvic radiographic parameters (PT, SS, PI) and LL/PI matching (LL–PI). TK was measured from T5 to T12, and LL from L1 to S1, using the Cobb angle method. Coronal balance was measured using CVA as the distance between the centre of the C7 body and the centre sacral vertical line. Sagittal balance was measured using SVA as the distance from the C7 plumb line to the superior posterior endplate of the S1. PT was measured as the angle subtended by a horizontal vertical line and a line connecting the centre of the femoral head to the centre of the upper endplate of S1 on lateral radiographs. SS was measured as the angle subtended by a parallel line from the upper endplate of S1 and a horizontal line on lateral radiographs. PI was the angle between the vertical bisector of the upper endplate of the S1 and a line connecting the centre of the femoral head to the centre of the upper endplate of S1 on lateral radiographs.

On the sagittal plane, positive values were used to indicate kyphosis of the thoracic spine and lordosis of the lumbar spine; negative values were used to indicate lordosis of the thoracic spine and kyphosis of the lumbar spine. The spinopelvic radiographic parameters were measured by two senior authors who were not involved in the surgical treatment and the average was documented.

The amount of immediate changes in spinopelvic radiographic parameters (Δ) was defined in the formula:

The amount of immediate changes in spinopelvic radiographic parameters (ΔSVA, ΔTK, ΔLL, etc.) = the scores of immediately post-operative parameters − the scores of pre-operative parameters.

### Assessment of quality of life Scores

Patients were evaluated pre-operatively and at the final follow-up (at least 2 years after surgery). The scores of quality of life were evaluated using two self-reported health-related questionnaires (ODI and VAS). The simplified Chinese version of the ODI version 2.1a was used [18, 19]. This ODI questionnaire contains ten sections: pain intensity, personal care, lifting, walking, sitting, standing, sleeping, sexual activity, social life, and traveling. Each subclass has six levels (a score ranging from 0 to 5): a score of 0 is used for best measured health, and a score of 5 is used for worst measured health. To calculate the level of disability, the sum for each section were used in the following formula: the ODI score = the sum/50 × 100%. The ODI score (from 0 to 100%) was used to determine the disability of the patients. The higher ODI score means the higher level of disability. A VAS (0 = no pain; 10 = unbearable) was used to evaluate pain intensity (back pain and/or leg pain). A senior spine surgeon was responsible for the distribution and recovery of the questionnaires.

The amount of mid-term improvement in quality of life (Δ) was defined in the formula:

ΔODI/ΔVAS = the scores of ODI/VAS at the final follow-up − the scores of pre-operative ODI/VAS.

The values of ΔODI and ΔVAS are negative, indicating that quality of life is improved and the greater the absolute value of negative value, the more obvious improvement of quality of life.

### Statistical analysis

Continuous data were expressed as mean ± standard deviation. Comparisons of the spinopelvic radiographic parameters and quality of life scores between pre-operative and post-operative was performed using paired *t* tests.

Linear regressions were performed to determine how much of the variations in ΔODI or ΔVAS could be attributed to the amount of immediate changes in spinopelvic radiographic parameters [20, 21]. The dependent variable in all models is ΔODI or ΔVAS. Univariate linear regressions were performed to analyse the relation between the amount of mid-term improvement in quality of life and the amount of immediate changes in radiographic parameters. Then, forward stepwise multivariate linear regression analysis was used to avoid multicollinearity [21]. This multivariate linear regression analysis was also used to identify independent factors. The regression equations were obtained by linear regression.

To further verify the predictive usefulness of the predictor(s) yielded by multilinear regression analysis for predicting ΔODI or ΔVAS, receiver operator characteristic (ROC) analysis was done to investigate cut-off values [20, 21]. The value of status variable (ΔODI/ΔVAS) is defined in the ROC analysis: a ΔODI who ≥ the mean values was defined as a weak ΔODI (0), while a ΔODI which is less than the mean value was defined as a strong ΔODI (1); a ΔVAS who ≥ the mean values was defined as a weak ΔVAS (0), while a ΔVAS who < the mean values was defined as a strong ΔVAS (1). When the sum of sensitivity and specificity is maximised, the corresponding predictor’s value is cut-off value. The area under curve (AUC) was a measure of the diagnostic power of the predictors. Two-sided *P* < 0.05 was considered as statistically significant. Data analysis was performed using SPSS 20.0 (IBM, Armonk, NY, USA).

## Results

### Demographic and clinical data

Seventy-five cases were finally included and were followed up for a mean of 31 months (range 24–50 months). There were 17 males and 58 females. Average age at the time of surgery was 59 years (range 50–70 years).

### Radiographic parameters and quality of life scores

**Fig. 1:**
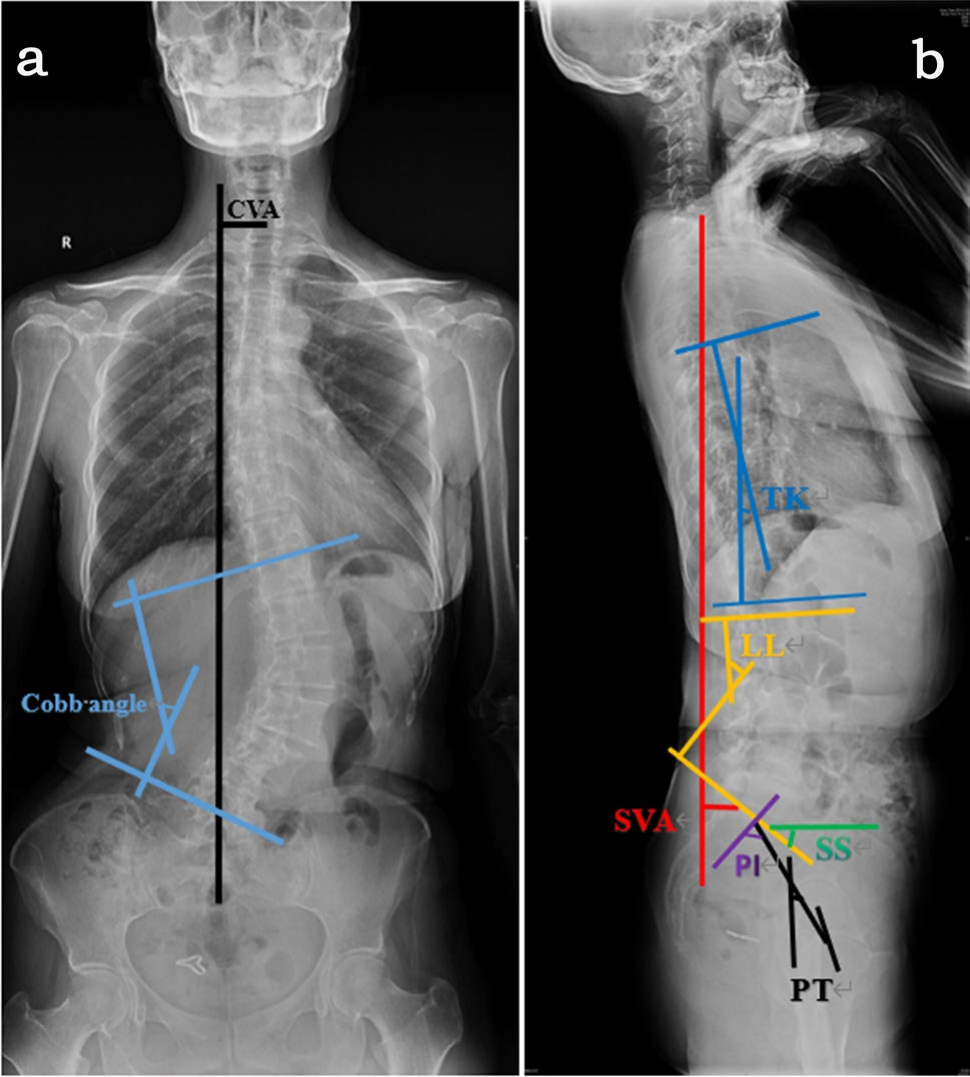
Measuring each pre-operative radiographic parameter in a 56-year-old woman with adult degenerative lumbar scoliosis. **a** measuring method of CVA (32.2 mm) and Cobb angle (42.5°) on anteroposterior radiograph. **b** measuring method of SVA (30.2 mm), TK (6.1°), LL (43.1°), SS (37.7°), PT (41.8°) and PI (79.5°) on lateral radiograph

**Fig. 2:**
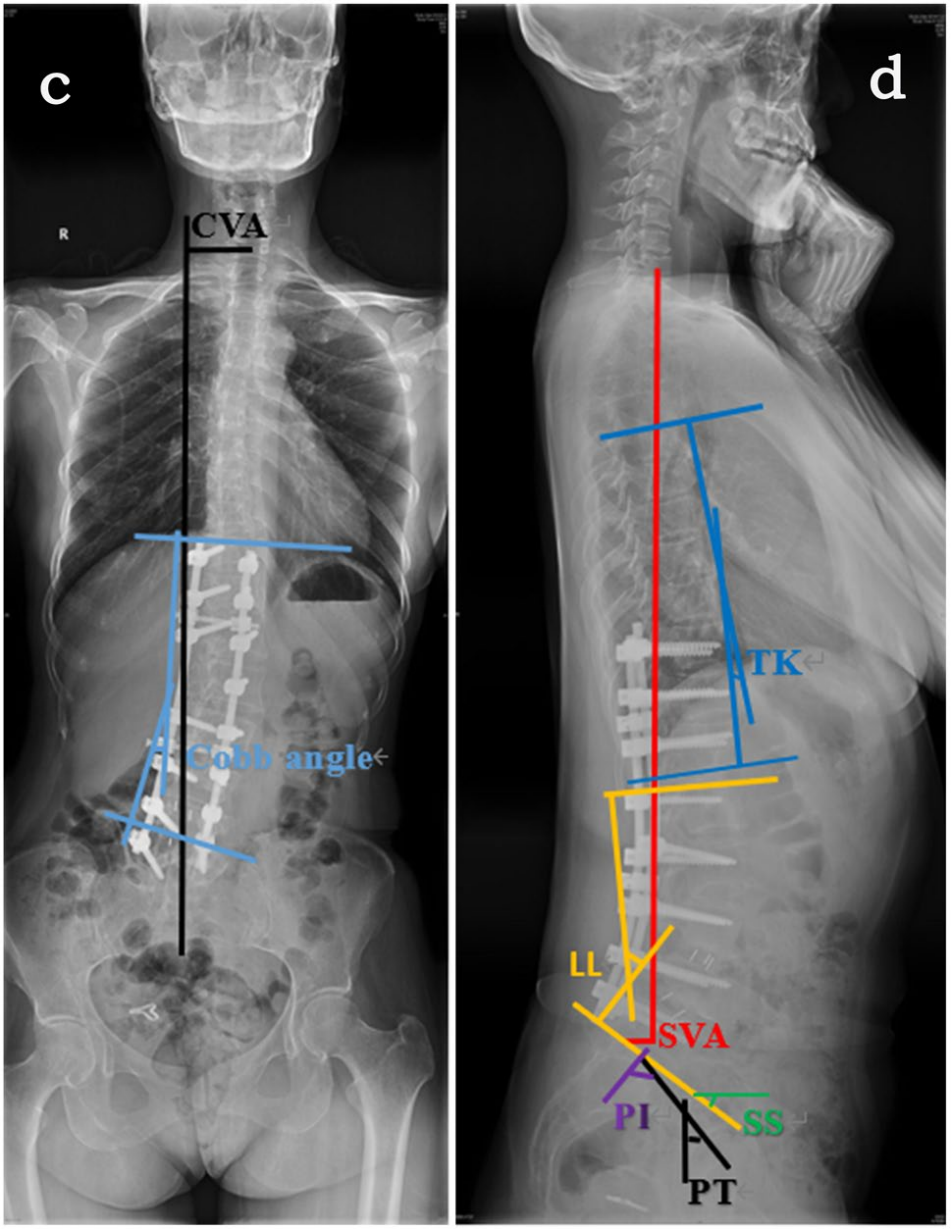
Measuring each immediately post-operative radiographic parameter in a 56-year-old woman with adult degenerative lumbar scoliosis. **c** measuring method of CVA (52.5 mm) and Cobb angle (12.4°) on anteroposterior radiograph. **d** measuring method of SVA (25.5 mm), TK (5.4°), LL (45.3°), SS (40.1°), PT (40.0°) and PI (80.1°) on lateral radiograph

Table 1 presents the spinopelvic radiographic parameters pre- and immediately post-surgery. Patients showed significant change in the Cobb angle (*P* < 0.001), SVA (*P* < 0.001), LL (*P* < 0.001), TK (*P* = 0.023), LL (*P* < 0.001), PT (*P* = 0.001), SS (*P* = 0.007) and PI–LL (*P* < 0.001), but there were no change in CVA (*P* = 0.152) and PI (*P* = 0.513). Figure 1 and Fig. 2 present a typical case.

**Table 1:** Radiographic parameters and quality of life scores

	Pre-operative	Immediate post-operative	Final follow-up	*P*	Change (Δ)
Cobb angle (°)	43.8 ± 19.0	21.2 ± 12.6	–	< 0.001*	− 22.6 ± 17.2
CVA (mm)	22.9 ± 16.2	19.6 ± 14.0	–	0.152	− 3.4 ± 20.2
SVA (mm)	46.5 ± 24.9	31.6 ± 15.4	–	< 0.001*	− 14.9 ± 20.7
TK (°)	31.3 ± 20.9	27.3 ± 14.9	–	0.023*	− 3.9 ± 14.7
LL (°)	26.1 ± 33.9	43.5 ± 13.3	–	< 0.001*	17.4 ± 26.3
PT (°)	17.1 ± 11.7	14.0 ± 11.7	–	0.001*	− 3.0 ± 7.5
SS (°)	27.0 ± 10.6	30.2 ± 9.7	–	0.001*	3.1 ± 7.7
PI (°)	44.1 ± 11.0	44.2 ± 11.0	–	0.513	0.1 ± 1.3
PI–LL (°)	18.0 ± 32.2	0.7 ± 15.1	–	< 0.001*	− 17.3 ± 26.3
ODI(%)	55.8 ± 12.7	–	31.8 ± 11.3	< 0.001*	− 24.0 ± 8.7
VAS	6.3 ± 1.3	–	2.9 ± 0.7	< 0.001*	− 3.3 ± 1.2

*Cobb angle* Coronal Cobb angle of major curve, *CVA* coronal vertical axis, *SVA* sagittal vertical axis, *TK* thoracic kyphosis, *LL* lumbar lordosis, *PT* pelvic tilt, *SS* sacral slope, *PI* pelvic incidence, *PI–LL* LL/PI matching, *ODI* Oswestry Disability Index, *VAS* visual analogue scale

*Statistically significant

At the final follow-up (Table 1), ODI and VAS were significantly improved compared with pre-operative values (both *P* < 0.001).

### Regression analysis of the amount of improvement in quality of life

Table 2 presents the standardised coefficients (*β*) of regression models of variables associated with ΔODI. Only ΔCVA (*P* = 0.045), ΔSVA (*P* = 0.001), ΔLL (*P* < 0.001), and ΔTK (*P* < 0.001) were associated with ΔODI using univariate linear regressions. Then, factors with *P* < 0.05 in the univariate analyses were included in the multivariate analysis [21]. To avoid multicollinearity, forward stepwise multilinear regression analysis was used. After using the multivariate regression, ΔSVA (*P* < 0.001) was significantly associated with ΔODI, but ΔCVA (*P* = 0.112), ΔLL (*P* = 0.864), ΔTK (*P* = 0.053) were excluded. So ΔSVA was an independent factor of ΔODI after using the univariate and multivariate linear regression analysis. A univariate linear regression model revealed that ΔODI had a linear regression with ΔSVA (Table 3). The equation for the correlation is shown as follows:

**Table 2:** The potential predictors using linear regressions

	ΔODI		ΔVAS	
	Univariate	First multivariate	Univariate	First multivariate
ΔCobb angle	0.046 (0.692)		0.108 (0.355)	
ΔCVA	0.232 (0.045)*	0.153 (0.112)	0.100 (0.395)	
ΔSVA	0.386 (0.001)*	0.392 (< 0.001)*	0.597 (< 0.001) *	0.633 (< 0.001)*
ΔTK	− 0.455 (< 0.001)*	− 0.209 (0.053)	− 0.386 (0.001)*	− 0.072 (0.439)
ΔLL	− 0.431(< 0.001)*	− 0.325(0.864)	− 0.355 (0.002)*	1.159 (0.478)
ΔPT	0.183 (0.116)		0.121 (0.300)	
ΔSS	− 0.180 (0.122)		− 0.103 (0.381)	
ΔPI	0.023 (0.844)		0.082 (0.482)	
Δ(PI–LL)	0.430 (< 0.001)*	0.031(0.987)	0.360 (0.002)*	1.566(0.339)

All results are presented as standardised regression coefficients (P-values)

*Statistically significant

*ODI* Oswestry Disability Index, *VAS* visual analogue scale, *Cobb angle* Coronal Cobb angle of major curve, *CVA* coronal vertical axis, *SVA* sagittal vertical axis, *TK* thoracic kyphosis, *LL* lumbar lordosis, *PT* pelvic tilt, *SS* sacral slope, *PI* pelvic incidence, *PI–LL* LL/PI matching

**Table 3:** A univariate linear regression model predicting ΔODI

*R*	*R*^2^	Adjusted *R*^2^	Std. error of the estimate
0.386	0.149	0.137	8.082

Model	UC		SC		
	*β*	SE	*β*	*T*	*P*
(Constant)	− 21.592	1.151		− 18.764	< 0.001*
ΔSVA	0.162	0.045	0.386	3.577	0.001*

*ODI* Oswestry Disability Index, *UC* unstandardized coefficients, *SC* standardised coefficients, *SE* standard error, *SVA* sagittal vertical axis, *β* standardised coefficients, *T* T value

*Statistically significant

$$\mathrm{\Delta ODI}=0.162\times \mathrm{\Delta SVA}-21.592.$$

Table 2 presents the standardised coefficients (β) from regression models of variables associated with ΔVAS. Only ΔSVA (*P* < 0.001), ΔLL (*P* = 0.002), and ΔTK (*P* = 0.001) were associated with ΔVAS using univariate linear regressions. Then, factors with *P* < 0.05 in the univariate analyses were included in the multivariate analysis together [21]. After using the multivariate regression, ΔSVA (*P* < 0.001) was significantly associated with ΔVAS, but ΔLL (*P* = 0.478), ΔTK (*P* = 0.439) were excluded. So ΔSVA was an independent factor of ΔVAS after using the univariate and multivariate linear regression analysis. A univariate linear regression model revealed that ΔVAS had a linear regression with ΔSVA (Table 4). The equation for the correlation was shown as follows:

**Table 4:** A univariate linear regression model predicting ΔVAS

*R*	*R*^2^	Adjusted *R*^2^	Std. error of the estimate
0.597	0.357	0.348	0.960

Model	UC		SC		
	*β*	SE	*β*	*T*	*P*
(constant)	− 2.828	0.137		− 20.683	< 0.001*
ΔSVA	0.034	0.005	0.597	6.365	< 0.001*

*VAS* visual analogue scale, *UC* unstandardized coefficients, *SC* standardised coefficients, *SE* standard error, *SVA* sagittal vertical axis, *β* standardised coefficients, *T* T value

*Statistically significant

$$\mathrm{\Delta VAS}=0.034\times \mathrm{\Delta SVA}-2.828.$$

### ROC analysis

**Fig. 3:**
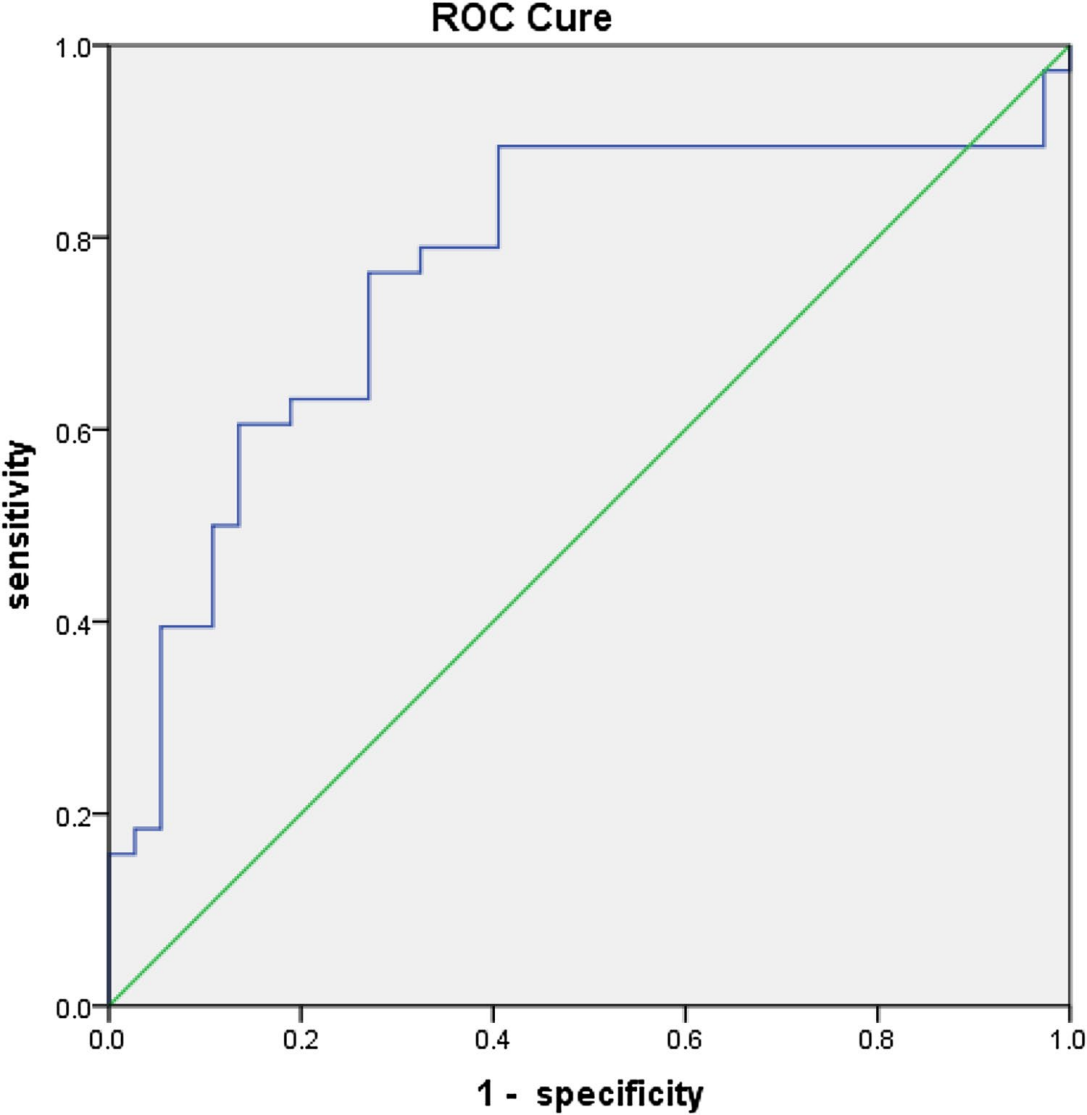
ROC curve for ΔSVA by ΔODI. Each point is a cut point for ΔSVA at which the sensitivity and specificity for predicting the weak ΔODI. *ROC* receiver operator characteristic, *SVA* sagittal vertical axis, *ODI* Oswestry Disability Index

**Fig. 4:**
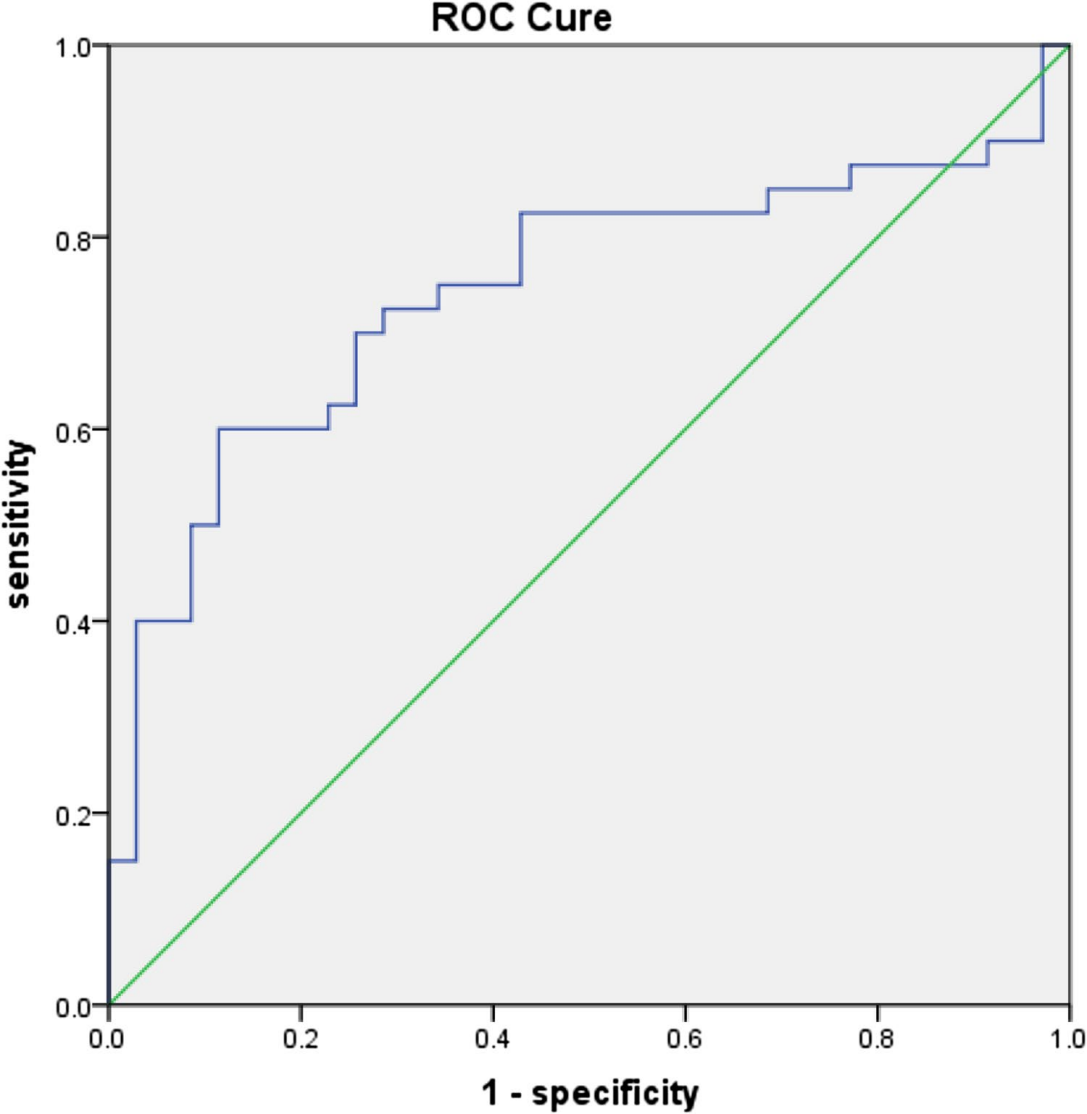
ROC curve for ΔSVA by ΔVAS. Each point is a cut point for ΔSVA at which the sensitivity and specificity for predicting the strong ΔVAS. *ROC* receiver operator characteristic, *SVA* sagittal vertical axis, *VAS* visual analogue scale

In the ROC curve for ΔSVA in the detection of a strong ΔODI, the cut-off value of ΔSVA was − 19.855 mm (sensitivity 76.3%, specificity 73.0%, AUC 0.767, *P* < 0.001) (Table 5, Fig. 3). Additionally, in the ROC curve for ΔSVA in the detection of a strong ΔVAS, the cut-off value of ΔSVA was − 15.405 mm (sensitivity 60.0%, specificity 88.6%, AUC 0.744, *P* < 0.001) (Table 5, Fig. 4).

**Table 5:** ROC curve analysis for ΔODI and ΔVAS

	ΔODI	ΔVAS
AUC	0.767	0.744
SE	0.058	0.059
95% CI	0.655–0.880	0.628–0.859
P	< 0.001*	< 0.001*
Cut-off value	− 19.855 (mm)	− 15.405 (mm)
Sensitivity	0.763	0.600
Specificity	0.730	0.886

*ROC* receiver operator characteristic, *ODI* Oswestry Disability Index, *VAS* visual analogue scale, *SVA* sagittal vertical axis, *AUC* area under curve, *SE* standard error, *CI* confidence interval

*Statistically significant

## Discussion

Some influential articles about adult spinal deformities also studied the various types together [4–7, 10, 12]. In addition, these articles only examined which indicators were relevant to quality of life [9, 10, 12], but there is no article quantifying the correlation between the amount of these indicators and the change of quality of life. To the best of our knowledge, this study is the first to directly assess this relation. Significant differences were seen between pre-operative and post-operative radiographic parameters in terms of Cobb angle, SVA, LL, TK, PT, SS, PI–LL, ODI and VAS. These data demonstrated that surgery was an effective treatment for ADS providing the more satisfactory spinopelvic radiographic parameters (except CVA and PI) in the immediately post-operative period and the better mid-term quality of life. By using univariate and stepwise multilinear regression analysis, we included the amount of the immediate changes in radiographic parameters and successfully identified ΔSVA as the independent predictor of the amount of the mid-term improvement in quality of life. Two predictive equations were yielded. Although the two equations could be used as predictive tools, the complexity of the coefficients may restrict their practicability in the actual clinical work. To simplify clinical practise, the cut-off values for ΔSVA were identified using ROC curves.

Sagittal balance has been confirmed to be an important radiographic parameter correlating with clinical outcomes of adult scoliosis [9, 22, 23]. Another study further showed that although mildly positive sagittal balance was somewhat detrimental, severity of symptoms increased in with progressive sagittal imbalance in adult spinal deformity [24]. However, these two studies [9, 24] chose a wider variety of patients with adult spinal deformity and beginning from 18 years old. On the contrary, Ploumis et al. [13] showed that the magnitude of SVA in patients without surgery (age over 50) did not demonstrate the significant correlations with clinical symptoms including ODI and VAS of ADS. A European multicenter analysis about symptomatic de novo degenerative lumbar scoliosis just showed that weak correlations were found between SVA and ODI (*r* = 0.296, *P* < 0.05) [25]. SVA ≥ 50 mm (Sagittal malalignment) has a negative impact on VAS score for low-back pain after decompression surgery in patients with lumbar spinal stenosis [26]. But using linear regression analyses, our study showed that ΔSVA was an independent predictor of ΔODI and ΔVAS. In addition, our study showed that ΔSVA were positively associated with ΔODI and ΔVAS. This implies that the amount of mid-term improvement in quality of life may be predicted by obtaining the amount of immediate change in SVA. These findings suggest that reduction of SVA (cut-off values: ΔODI: ΔSVA = − 19.855 mm; ΔVAS: ΔSVA = − 15.405 mm) may be important to achieve significant ΔODI and ΔVAS.

LL is also an important radiographic parameter correlating with clinical outcomes of adult scoliosis. A minimum 5-year follow-up study about ADS indicated a significant correlation between bigger post-operative LL and lower post-operative ODI [27]. To degenerative lumbar scoliosis, LL positively correlated with the lumbar function and the VAS for leg pain [2]. The present study also showed that ΔLL were negatively associated with ΔODI and ΔVAS using univariate linear regressions. But after using the multivariate regression, ΔLL were excluded. A study found that TK showed a significant, but rather weak (*r* = − 0.260, *P* < 0.05) correlation with pre-treatment quality of life in de novo degenerative lumbar scoliosis [25]. The role of ΔTK in predicting the amount of mid-term improvement in quality of life has not been studied for ADS in the previous studies. In the present study, though univariate regression analysis showed a significant correlation between ΔTK and ΔODI and ΔVAS, but multivariate regression analysis did not get a significant result. These indicated us that ΔLL, ΔTK might be the confounding factors for predicting ΔODI and ΔVAS. The reason may be that LL and TK will change with the change of SVA in corrective surgery [10, 28].

A study indicated that the magnitude of CVA in patients without surgery showed a negative correlation with vitality, and patients with coronal imbalance (CVA > 50 mm) showed worse physical function scores [13]. Moreover, the Cobb angle negatively correlated with VAS in the subjects with radiographic degenerative lumbar scoliosis [2]. However, most studies now support that coronal balance is not a key factor that affects quality of life in ADS [5, 6, 23], and this is similar to the results of this study. A comparative study found that there was no significant difference between the best and worst outcomes based on ODI for older patients (46–85 years old) in the Cobb angle of major curve and CVA [29]. The present study only showed that ΔCVA were negatively associated with ΔODI using univariate linear regressions. But after using the stepwise multivariate regressions, coronal parameters were excluded. The results of our study suggest that greater surgical correction of the amount of coronal parameters is unlikely to result in a significant ΔODI and ΔVAS.

In multivariate models for ODI, no significant association was observed between pre-operative ODI and pre-operative PT, while no significant association was observed between post-operative ODI and post-operative PT [10]. ΔPT, ΔSS and ΔPI were not significantly associated with ΔODI and ΔVAS in our study, but pelvic radiographic parameters play an important role in the restoration of sagittal balance. PT correlated with quality of life of adult spinal deformity, while high values of PT expressed compensatory pelvic retroversion for sagittal balance [30]. Higher pelvic incidence (PI) and PT are likely to lead to failure of sagittal realignment due to insufficient correction [31]. These results are different from this study. This may be due to the differences in research methods. We studied the correlation between the amount of immediate changes in PT, SS and PI and the amount of the mid-term improvement in quality of life, rather than the correlation between individual PT, SS and PI and quality of life score. PI is extremely important in spine sagittal correction [32]. PI determines the shape of the pelvis and affects other parameters, such as LL. PI is a pelvic anatomical parameter with individual differences and is relatively constant after bone maturity, representing the relative position of sacrum and femoral head. That is to say PI largely remains constant from pre-operative to post-operative. A PI critical value of 42° or lower is found to have a fourfold increase in the development of lumbar disc displacement and Modic changes [33]. Increasing PI is significantly correlated with more global sagittal imbalance in Ankylosing spondylitis patients with thoracolumbar kyphosis [34]. Schwab formula: LL = PI ± 9° [35]. It is not difficult to conclude that PI can be used to infer the ideal LL, which has guiding significance for the correction of LL [36]. The spine and pelvis will be discordant if LL–PI < 9°, and this discordancy is the basis of spinal imbalance and disease [37]. Proportional PI–LL is still of utmost importance to maintain optimal sagittal alignment and consequently improve clinical symptoms for adult spinal deformity [38]. Overcorrection of the LL was noted as significant risk factors of proximal junctional kyphosis after adult spinal deformity correction surgery [39]. Patients with adult spinal deformity surgery that received no implant prophylaxis and had sagittal overcorrection had the highest incidence of proximal junctional failure [40]. But one study showed that overcorrection (PI–LL ≤ − 10°) showed good surgical outcomes (ODI and VAS) without increasing proximal junctional kyphosis in degenerative sagittal deformity [41], the other study showed that Overcorrection was an effective treatment modality to maintain optimal sagittal alignment in patients with degenerative lumbar kyphosis [42]. The results of our study suggest that ΔPI–LL is unlikely to result in a significantly ΔODI and ΔVAS. This may be because we studied the correlation between ΔPI–LL and the amount of mid-term improvement in quality of life, rather than the correlation between post-operative PI–LL and post-operative quality of life score.

There are some limitations of this study. First, it was a retrospective study with its inherent biases and weaknesses. Second, these patients were enrolled at a single centre and treated by our own experience. Third, ΔODI and ΔVAS may be affected by many factors, but this study just focused on the immediate changes in spinopelvic parameters.

In summary, this study established the role of the amount of immediate changes in spinopelvic radiographic parameters in predicting the amount of mid-term improvement in quality of life of ADS with surgery. Reduction of SVA may be important to achieve a significant amount of mid-term improvement in quality of life. ΔCVA, ΔLL, ΔTK may be the confounding factors for predicting ΔODI and ΔVAS and the changes in the other radiographic parameters were not significant. Two predictive equations using ΔSVA with respective cut-off values may be useful to estimate ΔODI and ΔVAS.
